# Supplementary material for: Increased B Cell Selection Stringency In Germinal Centers Can Explain Improved COVID-19 Vaccine Efficacies With Low Dose Prime or Delayed Boost
Source: Front Immunol. 2021 Nov 30;12:776933. doi: 10.3389/fimmu.2021.776933 (PMC8669483; doi:10.3389/fimmu.2021.776933)
Supplement: Supplementary file 1 [file DataSheet_1.pdf]

Supplementary Material

1 Supplementary Figures and Tables

1.1 Supplementary Figures

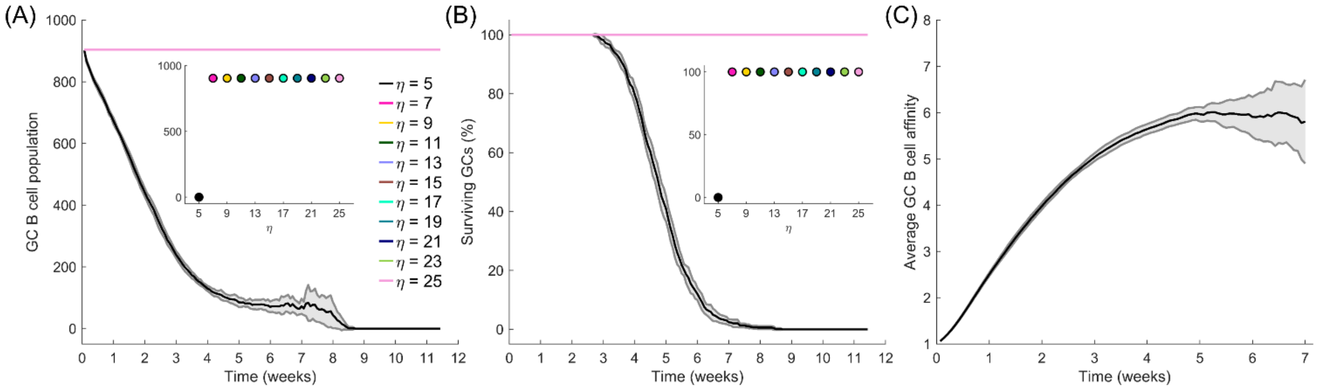

Supplementary Figure 1. Influence of η. Time series of (A) GC B cell population and (B) percent surviving GCs for simulations with various η. GCs with low η (=5 here) have high rates of apoptosis which result in GCs gradually being extinguished with time. However, these GCs also exhibit accelerated affinity maturation (C) due to the higher selection stringencies. Insets: values at the final time point.

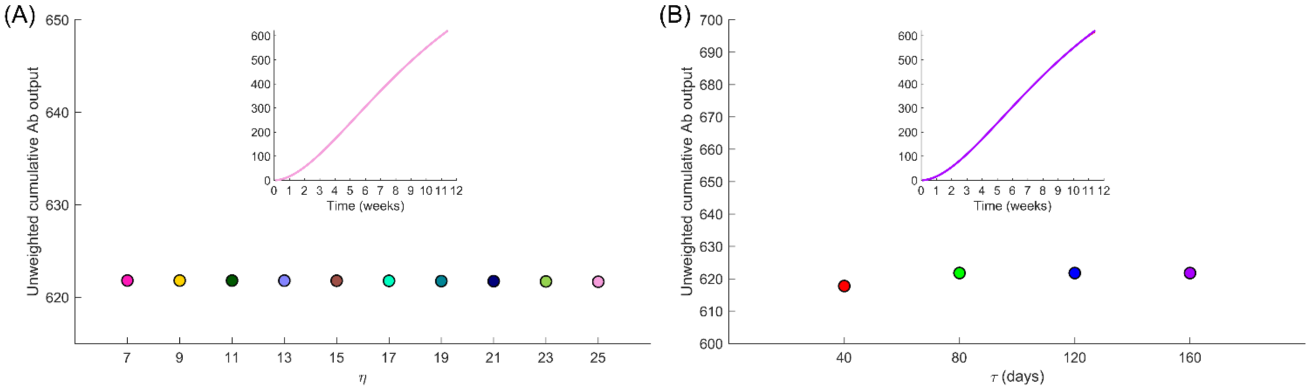

Supplementary Figure 2. Absolute Ab output. Unweighted cumulative antibody output at the final time point for various (A) η, and (B) τ. Insets: time series. The curves for the different cases overlap.

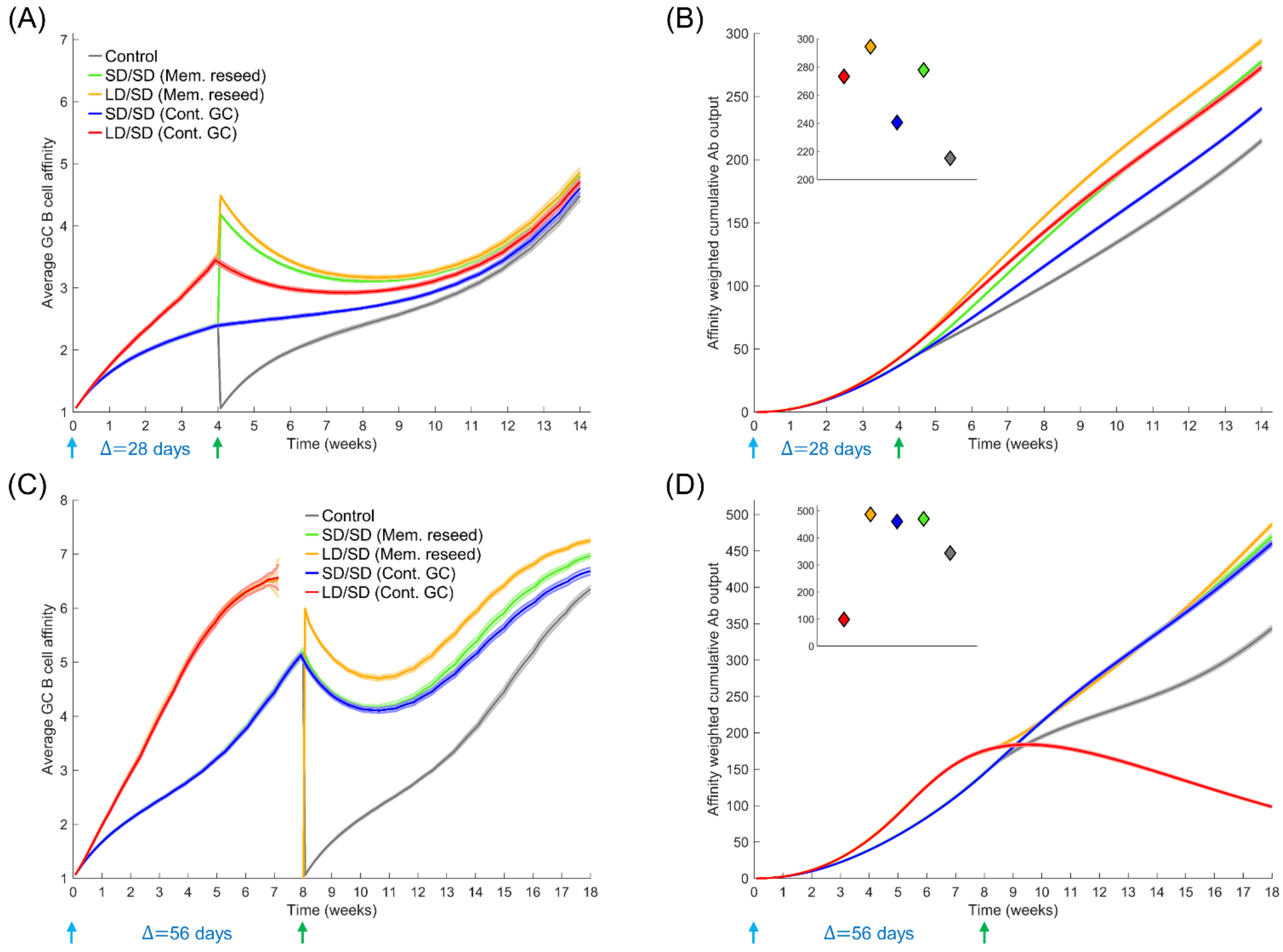

Supplementary Figure 3. Influence of initial Ag levels (dose) and the prime-boost dosing interval (Δ). (A) Average GC B cell affinity, and (B) affinity-weighted Ab output with a prime-boost interval Δ=28 d and antigen half-life τ=40 d, either with LD/SD or SD/SD dosing (LD and SD correspond to η_0=15 and 30, respectively). These trends are qualitatively similar to those in Figure 3. (C) Average GC B cell affinity, and (D) affinity-weighted Ab output with Δ=56 d and τ=40 d, either with LD/SD or SD/SD dosing (LD and SD correspond to the default η_0=10 and 20, respectively). Insets: values at the final time point.

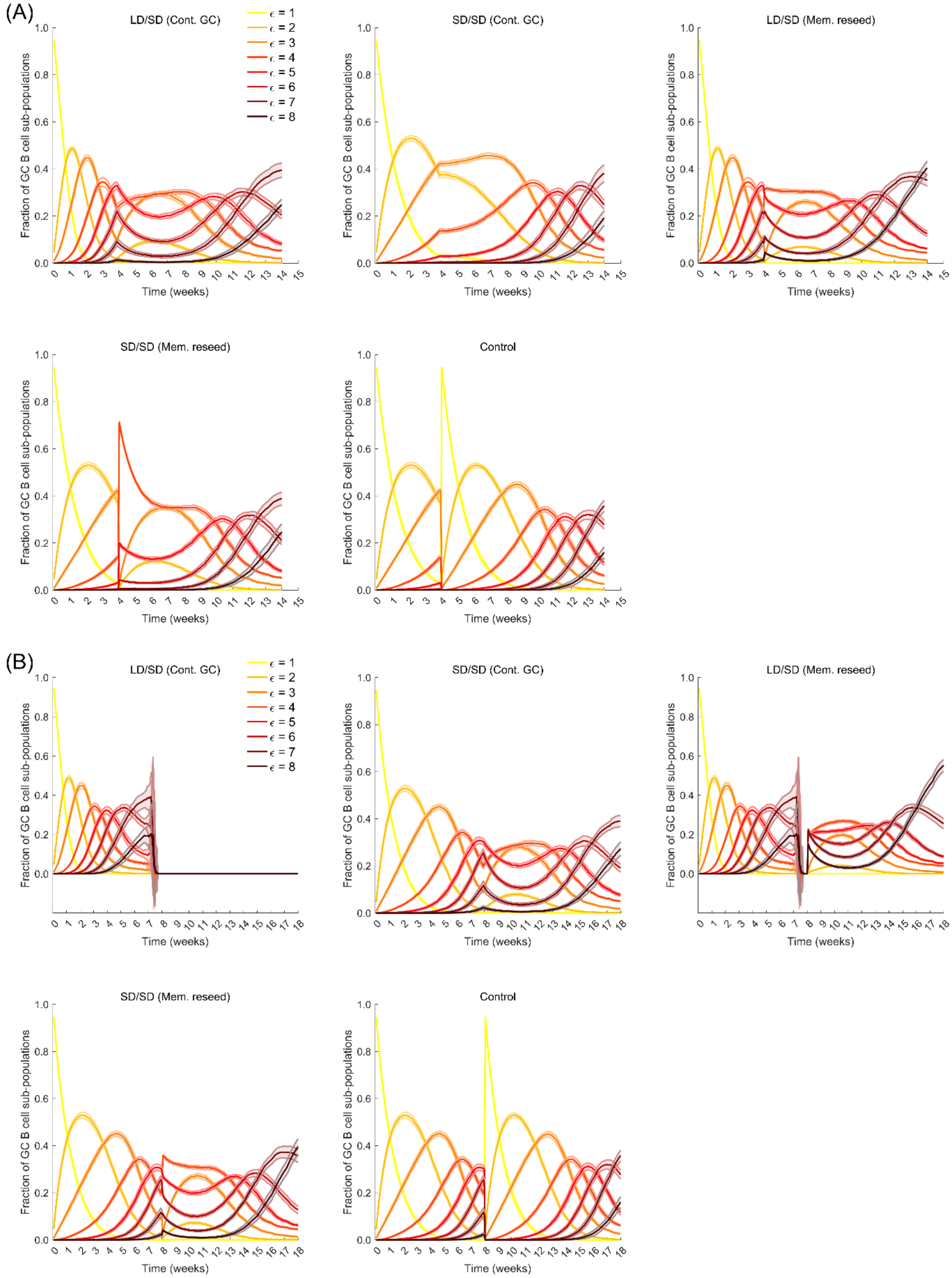
(A)
LD/SD (Cont. GC)
SD/SD (Cont. GC)
LD/SD (Mem. reseed)
SD/SD (Mem. reseed)
Control
ϵ = 1
ϵ = 2
ϵ = 3
ϵ = 4
ϵ = 5
ϵ = 6
ϵ = 7
ϵ = 8
Fraction of GC B cell sub-populations
Time (weeks)
(B)
LD/SD (Cont. GC)
SD/SD (Cont. GC)
LD/SD (Mem. reseed)
SD/SD (Mem. reseed)
Control
ϵ = 1
ϵ = 2
ϵ = 3
ϵ = 4
ϵ = 5
ϵ = 6
ϵ = 7
ϵ = 8
Fraction of GC B cell sub-populations
Time (weeks)

Supplementary Figure 4. GC B cell diversity upon vaccination. Time-evolution of populations of GC B cells of different affinity, ε, for vaccination with prime-boost intervals of (A) Δ=28 d, and (B) Δ=56 d, with antigen half-life τ=40 d, either with control, LD/SD, or SD/SD dosing (LD and SD correspond to η_0=10 and 20, respectively), and with continuing existing GCs or seeding using memory GC B cells following boost. (Colour scheme used is the same as Fig. 2A.)

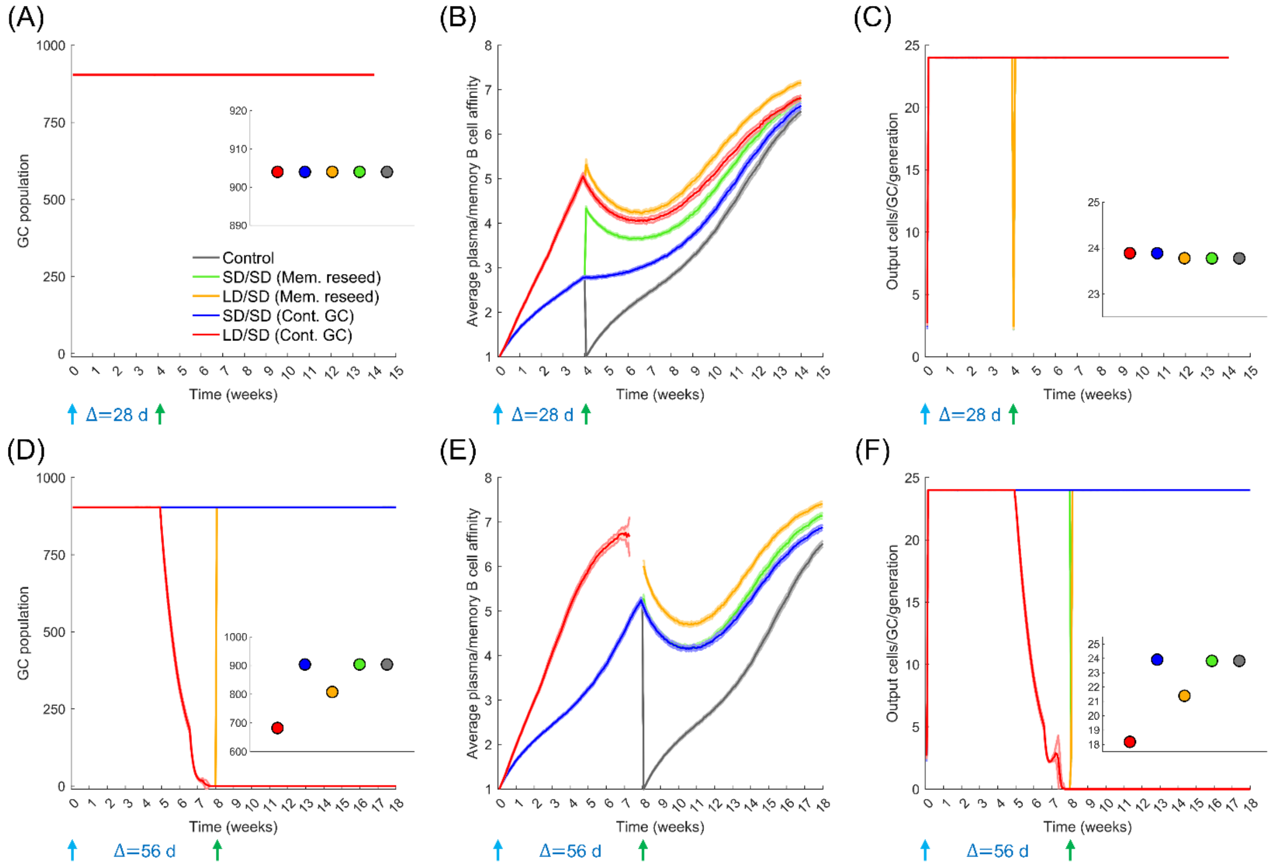

Supplementary Figure 5. GC B cell population and output upon vaccination. Time evolution of (A, D) the total GC B cell population, (B, E) the average affinity of output plasma or memory B cells, and (C, F) total output plasma and memory B cells per GC per generation upon prime-boost vaccination with Δ=28 d and 56 d, respectively. (τ=40 d; LD and SD correspond to η_0=10 and 20, respectively). *Insets:* averages over trajectories until GCs are terminated or maximum simulation runtime is reached.

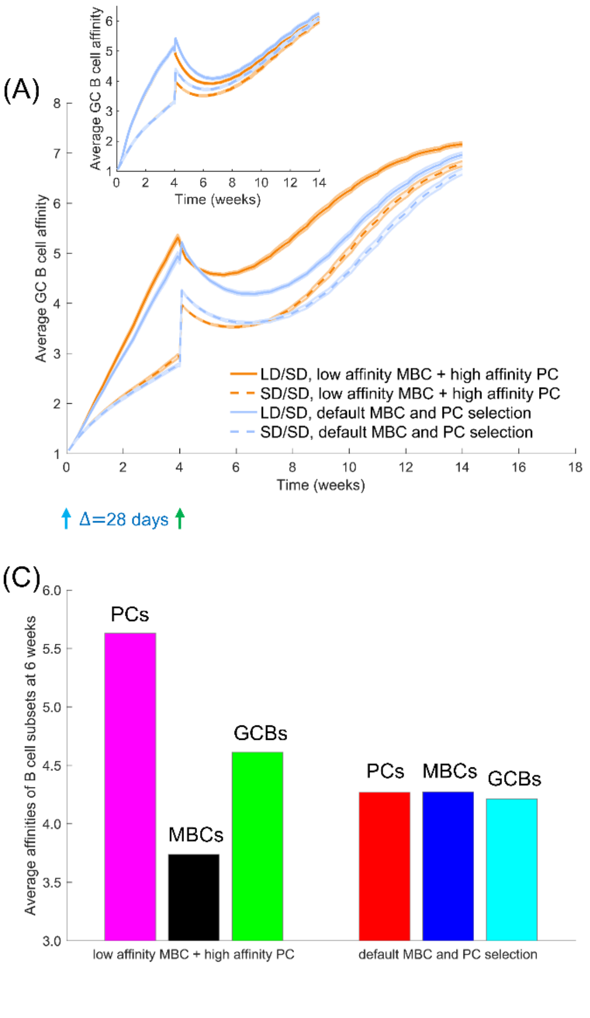

Supplementary Figure 6. Influence of the selection of memory B cells (MBCs) and plasma cells (PCs) in the light zone (LZ). Two B cell selection scenarios in LZ are compared: (i) default MBC and PC selection, where 10% of selected B cells in the LZ are equally and randomly divided between memory and plasma cell pools (Methods), and (ii) low affinity MBC and high affinity PC, where the selected 10% LZ B cells are sorted by their affinities and the top half differentiates into PCs while the lower half differentiates into MBCs. (A) Time series of average GC B cell affinities corresponding to Δ=4 weeks and with antibody feedback. Inset: simulation without antibody feedback (presenting Ab in the IC has a constant ω of 4). The low affinity MBC and high affinity PC scenario leads to higher quality Ab feedback and thus better AM, while this effect is lost when Ab feedback is removed (inset). The average affinities of PCs, MBCs, and GC B cells as a function of time (B) and at 6 weeks (C) with the two B cell selection scenarios. Low affinity MBC and high affinity PC selection leads to the formation of low affinity memory pool compared to PCs and GCBs. (D) Same as (A) but with Δ=8 weeks. Across both MBC/PC selection scenarios, the predicted trends remain qualitatively consistent: LD/SD results in higher B cell selection stringencies and thus higher GC affinities compared to SD/SD, and higher Δ leads to better AM. All simulations were performed with τ=40 d and with memory cells seeding GCs after boost.

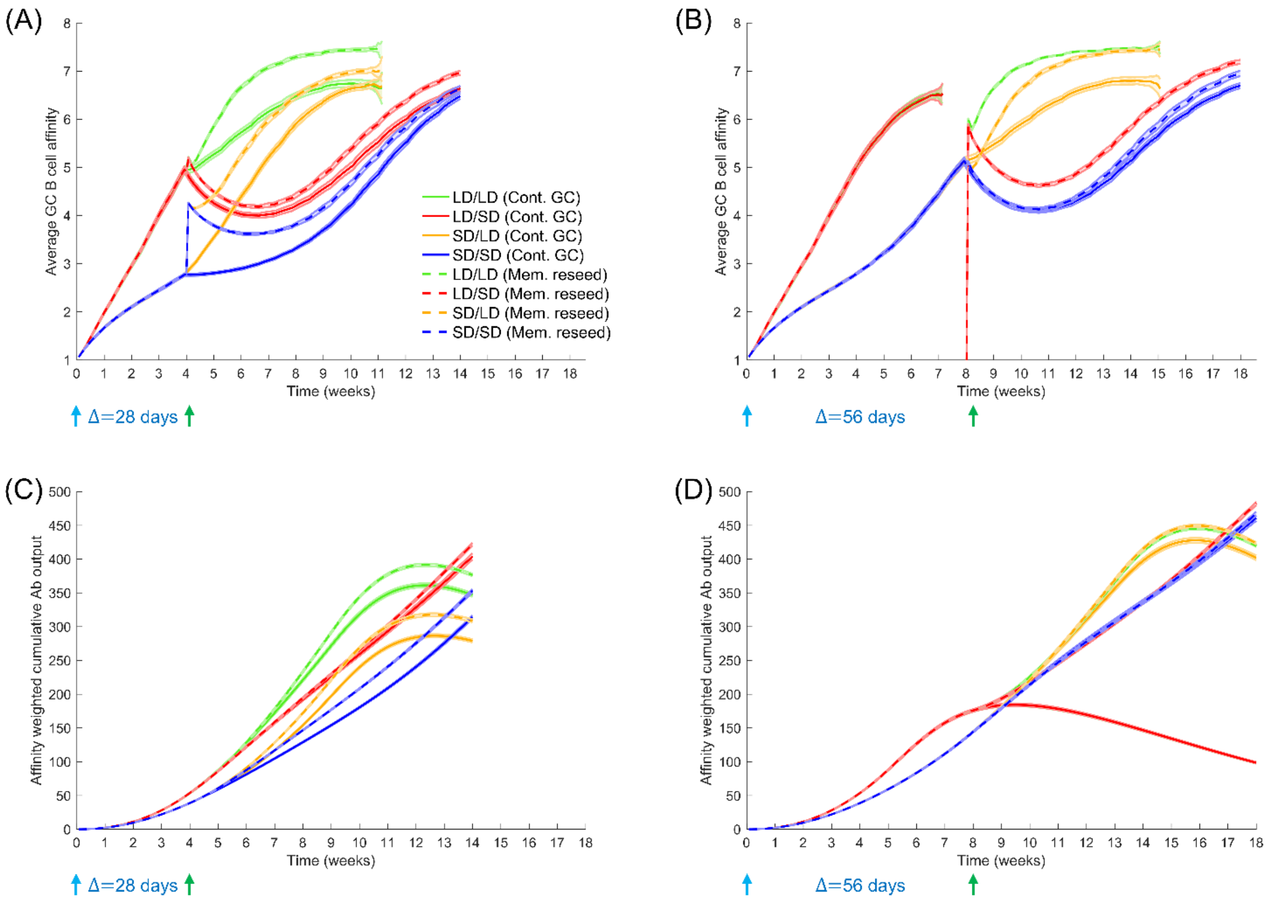

Supplementary Figure 7. Exploring the effect of alternative low dose-high dose combinations. (A, B) Average GC B cell affinity, and (C, D) affinity-weighted Ab output with prime-boost intervals of Δ=28 d and 56 d, respectively, and antigen half-life τ=40 d. Dosing scenarios explored include LD/LD, LD/SD, SD/LD, or SD/SD (LD and SD correspond to η_0=10 and 20, respectively).

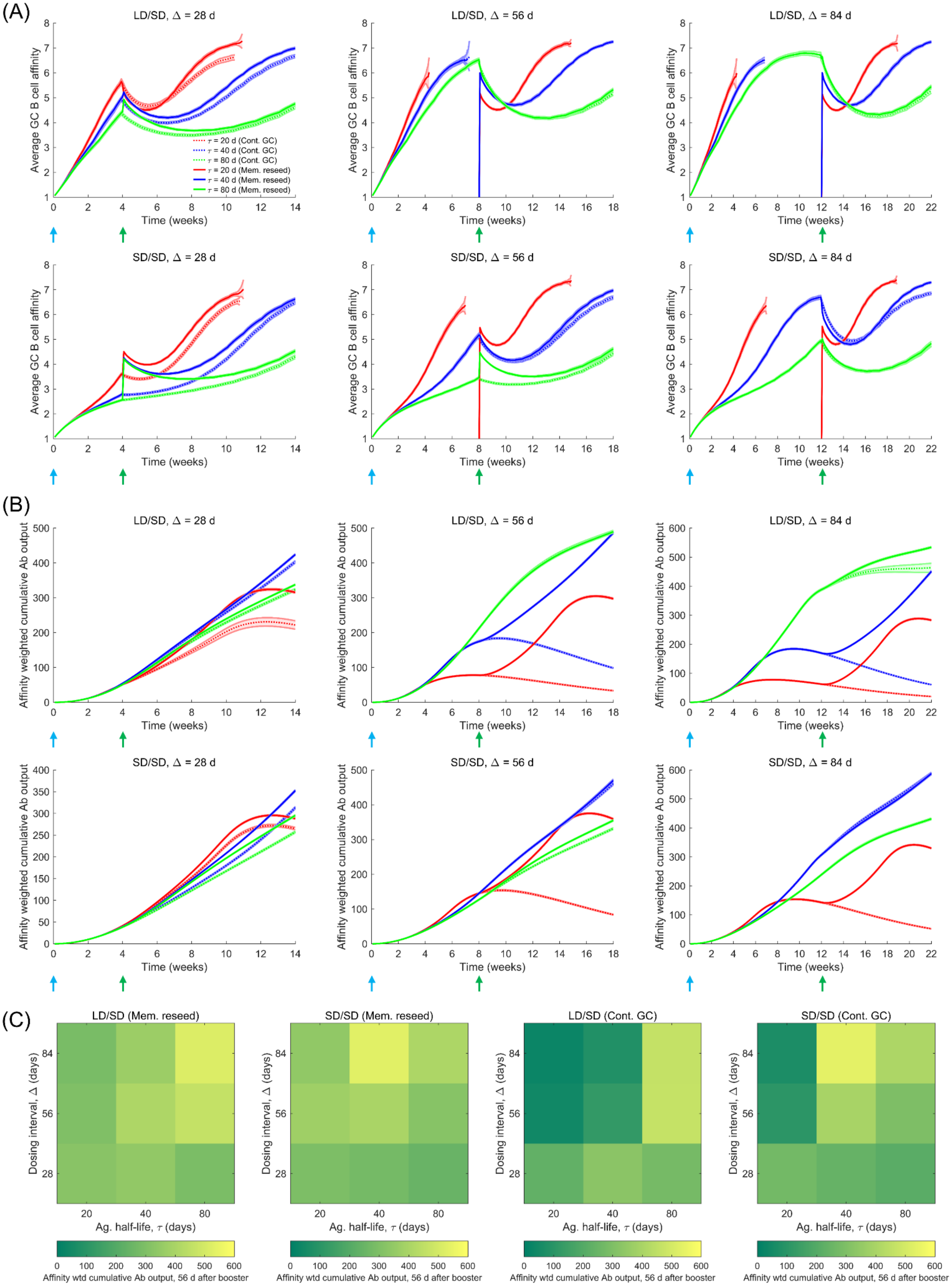
(A)
LD/SD, Δ = 28 d
LD/SD, Δ = 56 d
LD/SD, Δ = 84 d
SD/SD, Δ = 28 d
SD/SD, Δ = 56 d
SD/SD, Δ = 84 d
Average GC B cell affinity
Time (weeks)
τ = 20 d (Cont. GC)
τ = 40 d (Cont. GC)
τ = 80 d (Cont. GC)
τ = 20 d (Mem. reseed)
τ = 40 d (Mem. reseed)
τ = 80 d (Mem. reseed)
(B)
LD/SD, Δ = 28 d
LD/SD, Δ = 56 d
LD/SD, Δ = 84 d
SD/SD, Δ = 28 d
SD/SD, Δ = 56 d
SD/SD, Δ = 84 d
Affinity weighted cumulative Ab output
Time (weeks)
(C)
LD/SD (Mem. reseed)
SD/SD (Mem. reseed)
LD/SD (Cont. GC)
SD/SD (Cont. GC)
Dosing interval, Δ (days)
Ag. half-life, τ (days)
Affinity wtd cumulative Ab output, 56 d after booster

Supplementary Figure 8. Influence of the prime-boost dosing interval (Δ). Time series of (A) average GC B cell affinities, and (B) affinity-weighted Ab outputs corresponding to the heatmaps in Figure 4E and 4F, respectively. (C) Heatmaps of the affinity-weighted cumulative Ab output 56 d post the boost, as a function of τ (20, 40 and 80 d) and Δ (4, 8, and 12 weeks) for the two limiting scenarios (Mem. reseed and Cont. GC).

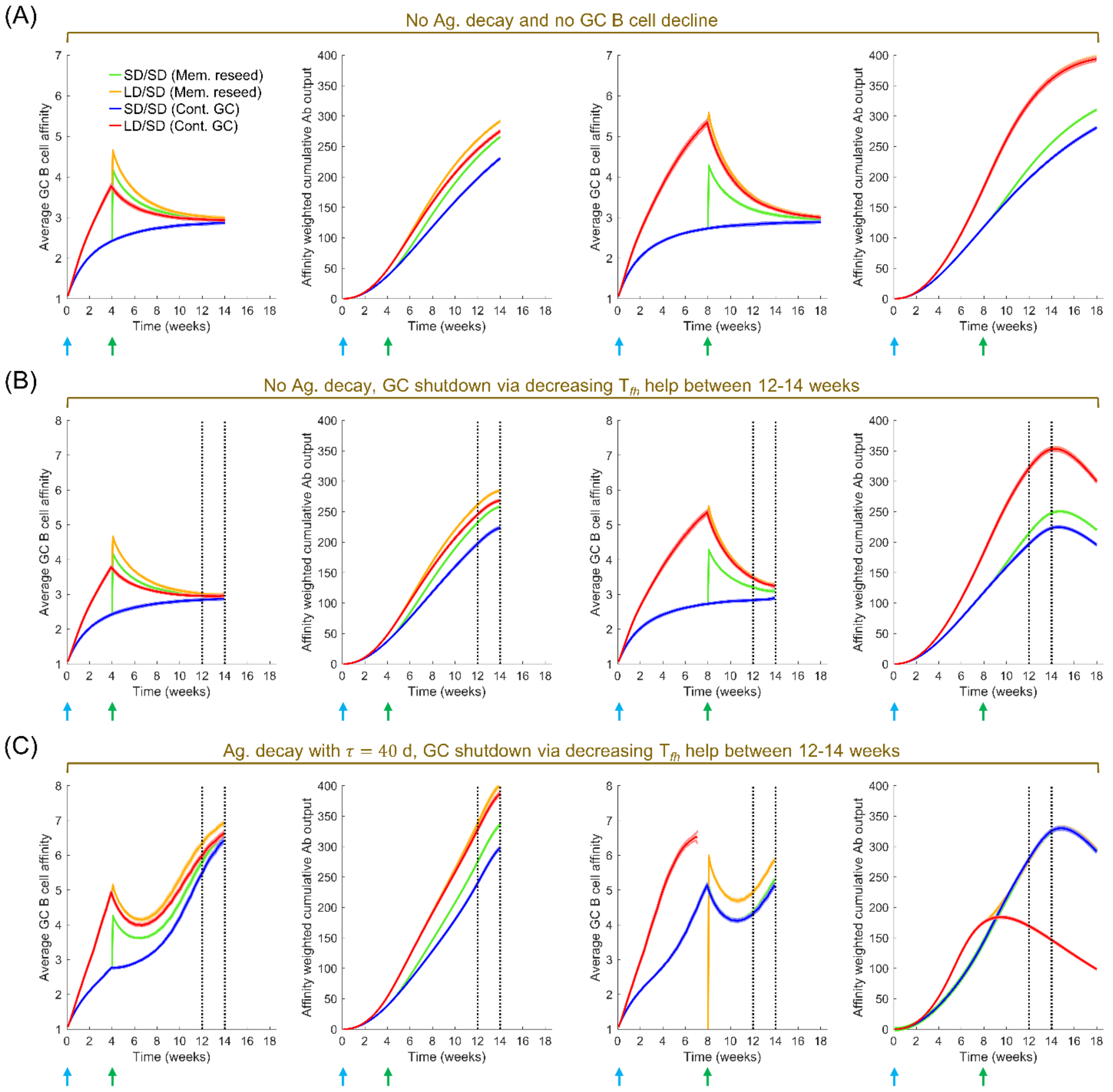

Supplementary Figure 9. Exploring alternative mechanisms of GC shutdown. Average GC B cell affinity and affinity-weighted antibody output from simulations with (A) no antigen decay and hence no GC shutdown due to declining B cell population, (B) No antigen decay but GCs are shutdown by linearly decreasing T_{fh} help between weeks 12-14 (the maximum number of GC B cells selected by T_{fh} cells linearly drops from 250 to 0 per generation), mimicking GC experiments, and (C) same as B but with antigen decay (antigen half-life, τ=40 d). Simulations were performed with prime-boost intervals of Δ=28 d and 56 d, respectively, and with either LD/SD or SD/SD dosing (LD and SD correspond to η_0=10 and 20, respectively). Our conclusions remained robust to the mechanism of GC shutdown; lower prime dose and delayed boost resulted in better GC responses.

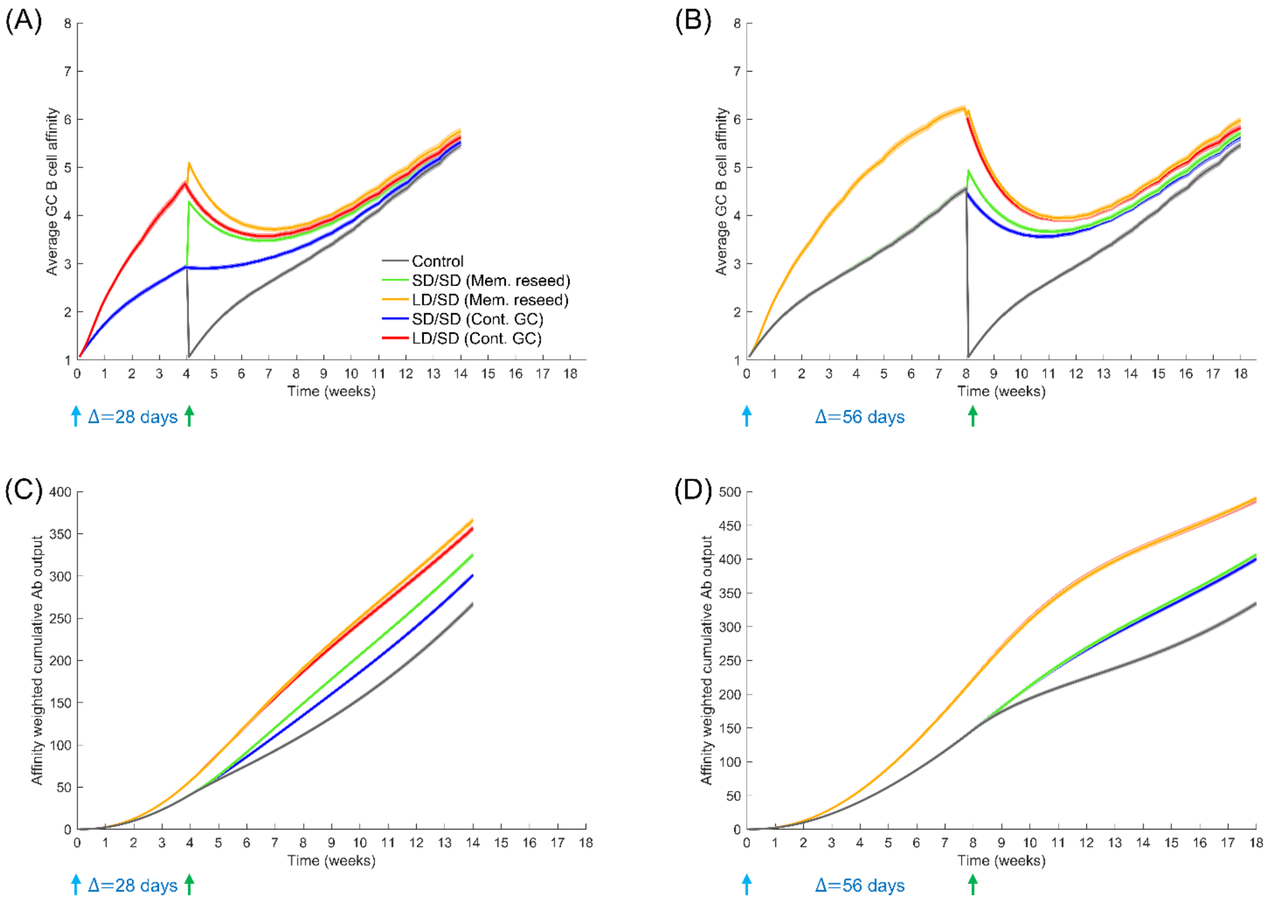

Supplementary Figure 10. Prime-boost simulations without antibody feedback. (A, B) Average GC B cell affinity, and (C, D) affinity-weighted antibody output with prime-boost intervals of Δ=28 d and 56 d, respectively, and antigen half-life τ=40 d, either with LD/SD or SD/SD dosing (LD and SD correspond to η_0=10 and 20, respectively), without antibody feedback (ω was fixed at a match length of 3; see Methods). Qualitative trends remained similar to simulations with antibody feedback (Fig. 3A, B), although affinity maturation was slower.

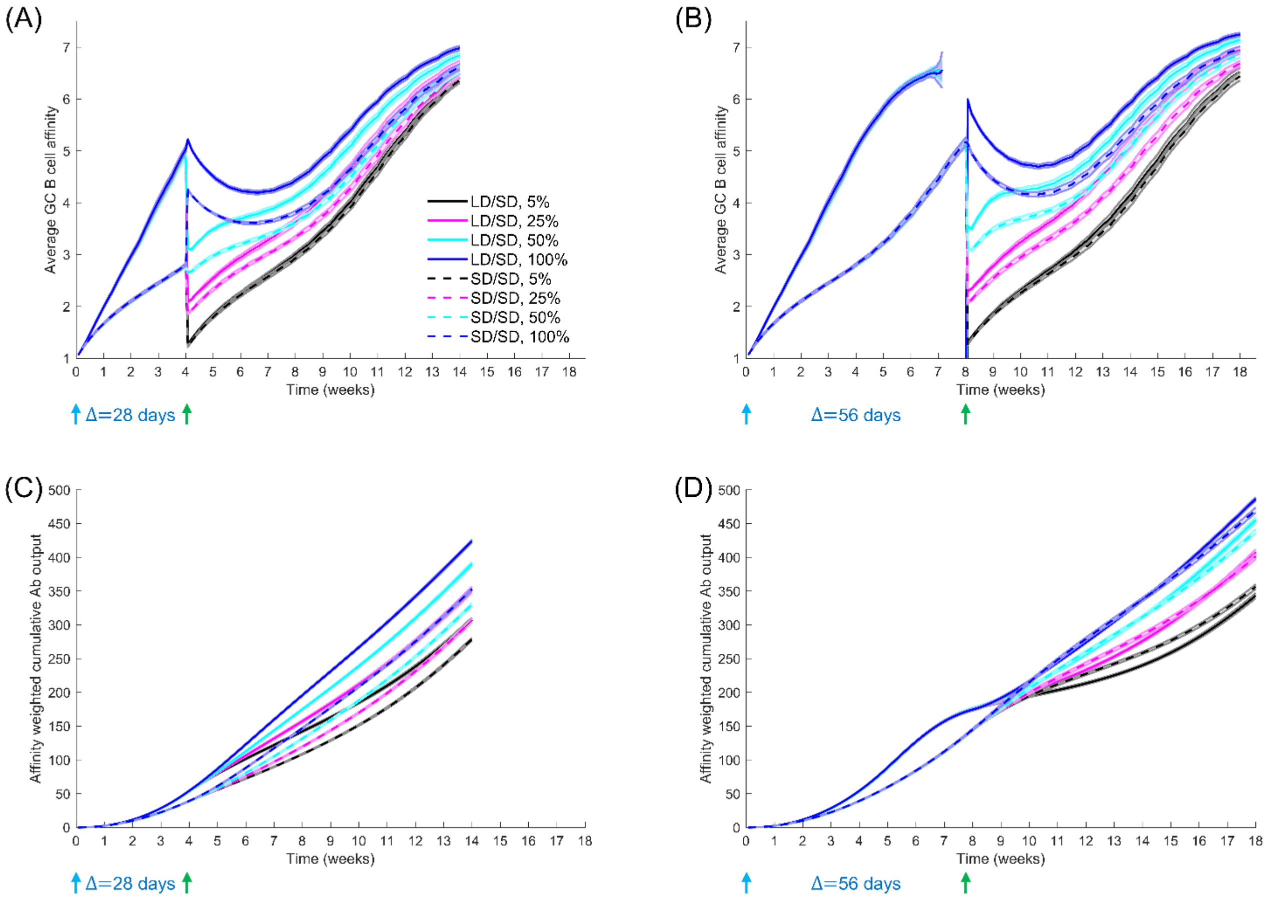

Supplementary Figure 11. Graded entry of seeder memory B cells into boost-induced recall GCs. (A, B) Average GC B cell affinity, and (C, D) affinity-weighted antibody output with prime-boost intervals of Δ=28 d and 56 d, respectively, and antigen half-life τ=40 d, either with LD/SD or SD/SD dosing (LD and SD correspond to η_0=10 and 20, respectively). Boost-induced recall GCs were seeded by 5%, 25%, 50%, and 100% (memory reseed scenario) memory B cells. Affinity maturation was observed to increase with higher fractions of seeder memory B cells. However, the qualitative trends remained unchanged; lower dose prime and a longer dosing interval induced better GC responses.

1.2 Supplementary Tables

Supplementary Table 1. Model parameters and their values.

Symbol	*Description*	*Value (units)*	*Refs.*
N	Number of B cells initiating the GC reaction	1000 (cells)	(36, 39)
L	String length of antigen, B cell receptor (BCR), and antibody	8 (dimensionless)	(39, 42)
κ	Alphabet size for strings	4 (dimensionless)	(39, 42)
θ_c	Minimum amount of acquired antigen for B cell survival	3 (dimensionless)	(39)
θ_∞	Maximum amount of antigen that can be acquired by a B cell	5 (dimensionless)	-
δ_p	Clearance rate of plasma cells	0.015 (generation^{-1})	(34, 44)
β	Antibody production rate of plasma cells per generation	2000 (molecules cell^{-1} s^{-1})	(88)
δ_a	Clearance rate of antibodies	0.01165 (generation^{-1})	(34, 44)
